# Perceptions of midwives on shortage and retention of staff at a public hospital in Tshwane District

**DOI:** 10.4102/curationis.v42i1.1952

**Published:** 2019-07-22

**Authors:** Mosehle S. Matlala, Thanyani G. Lumadi

**Affiliations:** 1 Department of Health Studies, School of Social Sciences, University of South Africa, Pretoria, South Africa

**Keywords:** midwives, midwifery practice, retention, shortage, staff, recruitment, working environment, job satisfaction, quality care

## Abstract

**Background:**

Midwifery is the backbone of women and child healthcare. The shortage of staff in maternity units is a crisis faced by many countries worldwide, including South Africa.

**Objectives:**

This study aims to explore the perceptions of midwives on the shortage and retention of staff at a public institution.

**Method:**

The study was conducted at one of the tertiary hospitals in Tshwane District, Gauteng Province. A total of 11 midwives were interviewed through face-to-face and focus group interviews. An explorative, descriptive generic qualitative design method was followed, and a non-probability, purposive sampling technique was used. Thematic coding analysis was followed for analysing data.

**Results:**

The impact of shortage of midwives was reported to be directly related to poor provision of quality care as a result of increased workload, leading to low morale and burnout. The compromised autonomy of midwives in the high obstetrics dependency units devalues the status of midwives.

**Conclusion:**

Midwives are passionate about their job, despite the hurdles related to their day-to-day work environment. They are demoralised by chronic shortage of staff and feel overworked. Staff involvement in decision-making processes is a motivational factor for midwives to stay in the profession. The midwives need to be in the centre of the decision-making processes related to their profession. The revision of the scope of practice and classification of midwifery profession away from general nursing complex by the South African Nursing Council (SANC) could place midwifery in its rightful status.

## Introduction

Midwives are important members of the healthcare team who contribute in ensuring that quality maternity services are rendered to patients. According to the *Pretoria News* newspaper (Hospital Maternity Staff Quit in Numbers 2016:1), it was reported that the selected public hospital in Tshwane District had an alarming turnover of midwives. The hospital serves as a referral institution providing the highest level of care for high-risk conditions. Besides other disciplines of healthcare services provided in this hospital, the focus of this research study was mainly on maternity healthcare services with midwives taking a central focus. The maternity services provided are high-risk antenatal, high-risk labour, postnatal and neonatal care. In this study, neonatal care was excluded as the main research question was on the maternity staff. The institution is a teaching hospital with doctors who have specialised in obstetrics and gynaecology, trainee doctors, midwifery students and other health disciplines. The student midwives placed to undergo continuous clinical training are from different nursing institutions and universities. The midwifery staffing of the units appears to be subminimum as there is evidence of consistent reliance on the agency staff to cover daily shifts.

## Literature review

Maternal and neonatal care is at the crux of every healthcare system. According to World Health Organization (WHO 2016a:1), there is a shown evidence that midwifery plays a vital role and is associated with improved quality care, and rapid and sustained reductions in maternal and newborn mortality. Poor maternal and newborn care is the determinant of maternal and neonatal morbidity and mortality (WHO 2016a:1). Midwifery has come to the fore because maternal and newborn health was made the focus of the Millennium Development Goals (WHO 2013:804).

Midwives play a key role in reducing maternal and neonatal mortality and are further regarded as the warriors on the frontline of healthcare, battling to ensure that women survive child birth and that babies are born safely, even in the most marginalised areas (WHO 2013:804).

In a study conducted by the WHO (2016a:10), it has been found that midwifery, including family planning and interventions for maternal and newborn health, could avert a total of 83% of all maternal deaths, stillbirths and neonatal death. Midwives who work within the framework for maternal and newborn care and within an enabling environment have the potential to bring care close to women and communities and tailor it to their social and cultural needs (Ten Hoope-Bender et al. 2014:6).

Shortage of midwives was found to be a problem in developed countries as well as in the middle-income countries (Pugh et al. 2012:498). The United Nations Population Fund (UNFPA 2014:14) report on State of the World’s Midwifery 2014 brings to light that midwives make up to 36% of the midwifery workforce across the 73 countries. Their specific contribution to the physiological process of normal birth and their high degree of focus on sexual reproductive, maternal and neonatal health (SRMNH) continuum of care makes them essential.

There is a global call for urgent investment in high-quality midwifery to prevent maternal and newborn deaths (Afenyandu, Adegoke & Findley 2017:1067). Barker (2016:826) asserts that the abnormal working patterns of midwives contribute towards them leaving the profession. In addition, Barker adds that midwives continue to go the extra mile through staying late and missing breaks to ensure that they get through what is often a heavy and challenging workload. For midwives, the ever increasing dedication and crisis management can seem to be the only response to rising workloads, whereas staffing deficits may contribute to poor clinical outcomes and may affect midwives’ health, morale and retention. Staffing levels models using birth rate plus have been adopted successfully in other European countries such as the UK, England and others to evaluate staffing needs (Yelland et al. 2013:579).

## Statement of research problem

The shortage of midwives in the public sector influences maternal care outcomes in a negative manner. Poor-quality workplace in health system weakens the ability of institutions to meet their performance targets and quality healthcare outcomes, and makes it more difficult to attract, motivate and retain staff. The researcher, during clinical accompaniment of the midwifery students, observed that many midwives are resigning which leads to shortage. Mentoring and supervision of students including the provision of quality care to mothers and babies in the maternity unit are affected, hence the need for the researcher to explore the midwives’ perceptions on shortage of staff and how the challenge can be addressed.

## Aim of the study

The aim of the study was to explore midwives’ perceptions on shortage and retention of midwives in a public hospital in the Tshwane District, Gauteng Province.

The objective was to explore the specific factors associated with the midwives’ intentions to stay or leave their primary employment in the public sector of Tshwane District, from the midwife’s perspective. The research question for this purpose was as follows: What are experiences and perceptions on the shortage and retention of midwives in the maternity wards of the public sector hospital?

## Research method and design

In this qualitative study, a generic inductive approach with a descriptive design was followed to explore the perceptions on the shortage and retention of midwives. A qualitative design investigates people’s reports of their subjective opinions, attitudes, beliefs or reflections on their experiences of things in the outer world (Percy, Kostere & Kostere 2015:78). Focus groups and individual face-to-face semi-structured interviews were conducted among participants.

### Setting

The study was conducted at a particular academic hospital in Tshwane District, Gauteng Province. Among other services, the hospital provides for high-risk maternal and neonatal care. The antenatal and postnatal wards have 21 beds each, while the labour ward consists of 6 labour beds, 3 admission beds and 10 high care beds. The institution is a teaching hospital with gynaecology and obstetrics registrars, student doctors and midwifery students. The student midwives are placed to undergo periodic clinical training from the nursing college and the two universities which are affiliated to the hospital. The midwifery staffing of the units appears to be subminimum as there is evidence of consistent reliance on the agency staff to cover daily shifts.

### Study population and sampling

The population comprised midwives in the maternity wards. Non-probability purposive sampling method was used to select 11 midwives working in the maternity units at the time and who fitted the eligible criteria of two or more years’ experience practising as midwives. The researcher included six midwives in the focus group discussions. Individual face-to-face interviews were conducted with five midwives, two of whom also took part in the focus group discussions.

### Pilot study

Data collection instruments were designed and tested on a different group of midwives at a different setting to evaluate the effectiveness of the tool. Interview guides were used and upon satisfaction on responses received, the tool was then confirmed to be ideal to be used in the main interviews.

### Data collection

Data were collected using an interview guide and a tape recorder. Midwives were interviewed during their working shift because of availability. A semi-structured interview with open-ended questions was used for the interviews. The interview guide comprised a wide range of open-ended questions, covering demographic factors, intentions of the midwives to either stay or leave the profession, reasons for their proposed intentions and perceptions on why other midwives opt for leaving. The same interview guide was used for focus group discussions and for individual interviews.

The researcher requested permission from the operations manager about the interviews and made appointments with midwives.

Data collection was started with one focus group discussion to get a broader perspective on the shortage and retention of staff, followed by individual face-to-face interviews to obtain in-depth data. Focus group discussion was performed to ensure that midwives can discuss the topic with other colleagues and for the researcher to obtain more information. Six midwives participated in the focus group discussion which was conducted by two interviewers. The first interviewer was there to ascertain that the discussions were conducted smoothly by supporting the participants in explaining the purposes and procedures of the interview and to obtain written consent from all participants. Because of the nature of business in the labour ward, the discussions were conducted in an empty labour room, so as to avoid moving the midwives away from their work stations.

Individual face-to-face interviews were conducted on a different day from the focus group. The researcher decided to conduct face-to-face interviews with a few midwives with the view that focus group may limit freedom of expression, especially with individuals who are not confident to voice their personal views in a group setting. Five participants were interviewed in a quiet office in their unit of work. The tape recorder was used to record all the information discussed. The research questions which were asked were the same as the ones used in the focus group discussions. Data saturation was achieved during the face-to-face interview with participant number three. Two more participants were interviewed thus confirming data saturation.

### Data analysis

Thematic data analysis was followed to analyse the data (Percy et al. 2015:80). After familiarisation with collected data, themes were generated. Data were summarised by category from each transcript and arranged according to themes. The themes were supported by quotations from the raw data and then compared and contrasted with existing relevant literature. Names and other identifiers were changed to protect the privacy of participants (Green & Thorogood 2014:72). The participants in the focus group were identified as Participant#, while the individual interview participants were identified as IP#.

### Ethical considerations

Ethical approval was granted by the Research Ethics Committee of the University of South Africa (HSHDC/567/2016) and the Faculty of Health Sciences of the University of Pretoria for the Gauteng Province (252/2017). Written permission to conduct the study was obtained from the Chief Executive Officer of the hospital as a selected site. Verbal permission was also obtained from the operations manager before conducting interviews in the maternity ward.

#### Confidentiality

Confidentiality means not disclosing information gained from research in other settings such as through informal conversations (Green & Thorogood 2014:72). Protection of confidentiality was achieved by limiting the persons who had access to taped materials. Only the researcher had access to taped materials during the research study process.

#### Autonomy

Harish, Kumar and Singh (2015:410) describe autonomy as the ability to decide for the self, free from control of others, and with sufficient level of understanding to arrive at a meaningful choice. The researcher explained the purpose and procedures of the research study, and participants were given an opportunity to read the information document before signing the consent forms. Consent forms were signed before the beginning of the interviews and group discussions.

#### Trustworthiness

Trustworthiness of this study was assured by using Lincoln and Guba’s criteria as cited in Anney (2014:276–278) of credibility, transferability, dependability and confirmability. A pretest of the data collection tools was done on midwives from a different setting of research site which was not included in the main study.

#### Dependability

According to Colorafi and Evans (2016:23), dependability is a component of trustworthiness, which can be fostered by consistency in procedures across participants over time through various methods of data collection. The researcher used semi-structured interviews with the aid of an interview guide. All participants were asked the same questions in the same order to ensure dependability.

#### Confirmability

Colorafi and Evans (2016:23) describe confirmability as reasonable freedom from researcher bias. Transparency in all the processes of the research study was portrayed through sharing the approach and methods of data collection with the ethics committees involved. The data collected were kept safe and would be available if needed by stakeholders.

#### Credibility

Grove, Burns and Gray (2013:199) state that internal validity (credibility) refers to the extent to which the effects detected in the study are a true reflection of reality, rather than the result of extraneous variables. The researcher did not alter the question, and the objectives were never altered throughout the research process. All participants were given the same opportunity to respond to the questions posed.

#### Transferability

External validity (transferability) is concerned with the extent to which the study findings can be generalised beyond the sample used in the study (Grove et al. 2013:202). For this study, generalisation was limited to only the population within the selected institution. According to Percy et al. (2015:79), external generalisation is not necessary, because the data are not quantifiable.

## Findings

Three themes which emerged from the data were shortage of midwives, reasons for leaving the profession and reasons for staying in the profession.

### Demographic data

The participant’s ages for both individual face-to-face and focus group discussions ranged between 24 and 50 years. Only four of the 11 midwives interviewed had a Diploma in midwifery and neonatal nursing science. The other midwives had a midwifery qualification as part of the basic R425 qualification as registered with the South African Nursing Council. Three midwives were at the time deployed in the high-risk antenatal ward, three in the postnatal ward and five in the labour ward. The midwives’ experiences in the maternity units ranged from 3 to 27 years, with the lowest years of experience allocated to the youngest (24) midwife in the group and the highest to the oldest (50) midwife in the group. All midwives interviewed were females as there were no male midwives working in the maternity units during the time of data collection.

### Categories and themes generated from the interviews

The categories and themes generated from the interviews are summarised in Table 1.

**TABLE 1:** Categories and themes generated from interviews.

Theme	Categories	Subcategories
Shortage of midwives	Impact of shortage	Increased workload Working overtime Poor quality of midwifery care Low staff morale, stress and burnout Lack of opportunity for training Increased utilisation of temporary employed midwives
Reasons for leaving the profession	Management-related issues	Lack of management support Fear for litigations Financial issues Lack of recognition and compromised autonomy Too much paperwork No flexibility on working hours
Reasons for staying in the profession	Professional and personal limitations	Fear for change Passion for midwifery No other place to go for midwives Availability of training opportunities and other resources

#### Theme 1: Shortage of midwives

The shortage of midwives seems to have a major impact on the delivery of service and the unpleasant working conditions of midwives. The midwives emphasised the negative impact of the shortage on their daily working experience. In this study, midwives cited an overwhelming limited number of staff per shift as a point of concern. Labour ward being the unit with more emergencies and unpredictable state of events is the one which needed more coverage in most cases. To balance the shift, some midwives placed in antenatal and/or postnatal wards are requested to assist in the labour ward. That according to the affected midwives leaves a gap in their respective units of allocation.

**Increased workload:** Labour ward is seen as the unit that carries more workload compared to other maternity units by some of the midwives who are working in other parts of maternity section such as postnatal and antenatal wards; hence, some dread to work in the labour ward. In the study, midwives reported that the poor control of referral system is an element which causes extensively high workloads as they work in a referral hospital. The high workload was also caused by low-risk patients from the low level of care who are attended before being redirected to the relevant level of care.

‘…We are supposed to be rendering service to clients with high risk perinatal conditions but we end up with low risks.’ (#6FG, 43 years old, advanced midwife)

**Working overtime:** Overtime is commonly performed for reasons of income augmentation. It is however found not to be the only reason stated by midwives in the study. The midwives expressed their reasons for performing overtime as being to meet the unit’s demands. The overtime was performed in the same units in which they are normally deployed. Working overtime in the same unit where normal shift is done extends the burden of workplace stressors according to the midwives, because of the fact that the reward is different but the environment remains unchanged unlike with the agency midwives.

**Poor-quality midwifery care:** Midwives alluded that they are doing the best in providing midwifery care, but they are aware that sometimes it is of substandard state, explaining the reasons as related to the influx of patients from all over choosing to come to their institution as it is by far the best hospital in the area as compared to other public institutions.

‘Shortage of staff affects the quality care we aim to deliver….’ (#3FG, 50 years old, advanced midwife)

**Low staff morale, stress and burnout:** The midwives predominantly reported low staff morale as a factor which could contribute to them leaving. They report a sense of devalue by doctors and managers.

‘Coming to work knowing that we are short for a day is really discouraging, delegation of duties is predictable. You may work in high care section for successive days and it’s tiring.’ (IP#4, 26 years old, midwife)

**Lack of opportunity for training:** The midwives expressed their concern about not having an opportunity to attend workshops and symposia. They mentioned the importance of training and refresher courses to enhance their knowledge capacity. The midwives cited that they appreciated the opportunity to advance their careers as qualified advanced midwives, but wished that they could also be allowed to attend the workshops and relevant symposia. The situation of chronic shortage of staff deprived them of that opportunity.

‘We do not get the opportunity to attend any workshops outside the institution, for example, the sensitive midwifery symposium is hosted every year but we are never released to attend….’ (#4FG, 43 years old, advanced midwife)

**Increased utilisation of temporary employed midwives:** Utilisation of temporary employed midwives is implemented as a remedy to the chronic shortage of staff in the maternity unit. The midwives appreciate the effort to relieve them of workload through agency staff but reiterated their concern over the solution. They reported that temporarily employed staff need to be orientated and supervised despite being qualified midwives because they do not know the culture and the vision of the institution. The midwives also mentioned that the ideal staffing need for the labour ward is seven midwives per shift, further explaining that only three permanent midwives usually cover the unit.

#### Theme 2: Reasons for leaving the profession

Lack of management support, issues related to management, fear for litigation, financial issues, lack of recognition and compromised autonomy, too much paperwork and lack of flexibility in working hours were cited as reasons for leaving the profession.

**Lack of management support:** The support from the managers is perceived to be non-existent. Maternity section is seen as an environment where survival is achieved through resilience. The participants explained that working in the maternity ward, especially the labour ward, with poor or lack of management support, makes it more difficult, as it is an area which is unpredictable. They believe that managers have the responsibility to ensure worker wellness. The participants, however, reported one of the benefits of working in this institution as the availability of the opportunity to further their studies through study leaves.

‘Sometimes I feel like managers are on a “witch hunt”, they always find fault, never a good thing. It is like “us” against “them”.’ (IP#4, 26 years old, midwife)

The participants commented that there is lack of appreciation or understanding that managers have about the difficulties they face in their work environment. Adequate support would encourage them to work harder, without harbouring intentions to leave. The midwives noted the fact that they are aware that shortage of staff is a national crisis, but believe that migration is the better way of relieving the pressures on work issues. The midwives perceive the managers as people who stay in offices with no knowledge of the context of the midwives’ work environment with the authority to design policies and impose on them. They feel excluded from policy-making and changes made about their environment.

‘If management could show appreciation to the midwives, it would mean a lot. When you see them in the ward you know something is wrong. They never say anything positive. They are always criticising and it’s frustrating. This is the reason why there is a great exodus of the midwives. Appreciation is not only in the form of money.’ (#4FG, 43 years old, advanced midwife)

**Fear for litigation:** Although the midwives in the study expressed that their independent practitioner status is compromised, they still fear litigation, which could result from poor-quality care, delay in patient management and mostly poor recording while waiting for the doctor to make decisions to care for complicated cases. The midwives perceived the reasons why other midwives are disinterested in practising midwifery relating to their fear of litigations.

‘Midwifery is a risky profession. There is so much pressure from management and the community. There are so many litigations. This is the reason why many students would not choose to practice as midwives. It is so easy to lose your epaulets.’ (IP#1, 24 years old, midwife)

**Financial issues:** The midwives emphasised the need to be remunerated accordingly in relation to their qualifications and experience. The midwives mentioned that they are promoted with 10 or more years of work experience and that their salary levels are the same as those who have just qualified, and specialised in midwifery.

‘… you stay on the same salary scale for a long time. We are expected to wait for 10 years in a speciality unit in order to be promoted to the next rank.’ (#3FG, 50 years old, advanced midwife)

**Lack of recognition and compromised autonomy:** The midwives in the study raised a concern that their independent function as midwives is ignored by doctors, emphasising that most of the caesarean sections maybe avoided if midwives could be given a chance to showcase their expertise and experiences.

‘… this is a teaching institution; the students will never be able to witness a twin delivery because once diagnosed during pregnancy, doctors just book the woman for caesarean section.’ (IP#3, 25 years old, midwife)

‘The independent responsibilities of midwives are interfered with, only medical doctors have a complete say, midwives are expected to follow orders.’ (#5FG, 31 years old, midwife)

**Too much paperwork:** There is an extensive amount of administration attributed to midwives as they expressed their frustrations over the unnecessary documentation which is in most cases a repetition of information. An example of the obstetric book which is commonly used as a standard comprehensive perinatal care document, supplied to the facilities by the national department of health, was pointed out. Inside the book, there is also a provision for the consent form for caesarean section and notes if required. Different charts are introduced to be used in the high care, such as intensive care unit (ICU) patient charts. This increases the amount of paperwork the midwives need to deal with.

‘The standardised obstetric book was supposed to make our work easier by compressing all information regarding the perinatal care of the woman. Now there are many papers which we use outside the book, which are a duplicate to the book itself. It is so frustrating in this state of shortage of staff.’ (#5FG, 31 years old, midwife)

**No flexibility in working hours:** Midwives indicated that even though the conditions of employment allow all pregnant staff members the right for 4 months of maternity leave, it is difficult coming back after leave having to work a night shift when breastfeeding needs to continue. All staff members book for night duty in the beginning of the year, and when their turn comes, they are not ready because of personal circumstances. It is the responsibility of a concerned staff member to find a colleague who is willing to swap the shift. Working hours in maternity wards are rigid with a depleted workforce, where participants have little to suggest in terms of improvement.

‘I feel like we spend a lot of time here at work. I wish they could just include the time spend taking report and handing over within the paid working hours. Also the 40 hours per week is too much because our shifts are 12 hours long, if it could be reduced to 30 hours per week, it’s hectic!’ (#6FG, 43 years old, advanced midwife)

#### Theme 3: Reasons for staying in the profession

‘the independence nature of midwifery is what attracted me to the profession…despite all the negative experiences, I love what I do…seeing a successful case from admission to discharge gives me the satisfaction.’ (#1FG, 24 years old, midwife)

Similar to the reasons for leaving, the intentions to stay are multi-faceted depending on the circumstances around the individuals. Decisions as to whether to move or not did not affect midwives as individuals, but the entire family depends on where they ought to be moving to.

**Fear for change**

‘I believe that most midwives who don’t leave this place is because they have fear of the “unknown”. Midwifery is all they know; they would rather stay here because there is no other choice.’ (IP#4, 26 years old, midwife)

Change for some is a monster, and they would rather stay where they are conversant with the culture, than face new challenges. Older participants had expressed hope that the current situation would change, believing in resolving the problem rather than running away from it. The midwives compare their current employer to others on the same level of care, reporting that their work conditions are far better than the rest.

They reported the availably of material resources such as medicine, linen, stationery and world-class equipment for maternity care, such as cardiotocograph (CTG) machines, as a positive factor that attracts them to stay.

Some midwives expressed their fear of change related to the potential obligations to relocate or travel further than their current place of work. While changing jobs could be positive, it can also be taxing on an individual. Midwives have organisational loyalty, especially for those who received their further career development through paid fulltime study leave granted by the institution.

**Passion for midwifery:** The participants perceive midwifery as the best choice they made. It gives them fulfilment, despite the other negative aspects attached to it. Although there are challenges, the midwives do not plan to leave the practice completely, but plan to leave for better opportunities. The younger midwives have a wider vision of opening their own private midwifery practices or well-baby clinics. Midwives in the study expressed their passion for midwifery, hence the intention to stay in the profession. They explained that they find satisfaction in the successful delivery and seeing the mother and baby discharged in a healthy condition.

‘I have a passion for midwifery, as much as there are many challenges here I do not see myself practicing anything else.’ (#1FG, 33 years old, advanced midwife)

‘I think nurses choose different disciplines because of passion. If you don’t “click” with midwifery when you are still a student, it’s very uncommon to like it after qualification. Most registered nurses have a midwifery bar because of the comprehensive course that came as a package.’ (#1FG, 33 years old, advanced midwife)

**No other place to go for midwives:** Midwifery is all they know according to the work experience they have, because they have obtained their midwifery qualification. The midwives have developed workplace resilience. The older midwives reported that they have hope for the future of midwifery, but did not share the intentions to either move to another institution or leave the profession completely. The midwives cited that all the public institutions face the same challenge of shortage of staff and material resources

Leaving this institution will not help, shortage of staff is everywhere. (#3FG, 50 years old, midwife)

**Availability of training opportunities and other resources:** The participants appreciated the fact that the institution is a teaching institution for all health cadres. Being part of such important multidisciplinary teams, not only in the province, but in the country, gave them a sense of insight. The midwives who were not trained for a speciality in advanced midwifery mentioned that they do not see the difference between themselves and the advanced midwives, as they are exposed to similar learning opportunities. The doctor’s rounds with the obstetric consultants and other multidisciplinary teams were highlighted as highly beneficial to their professional growth. They also mentioned that most midwives in the unit are already trained as advanced midwives, opening opportunities to newly-qualified midwives not to wait long for their turn to train.

‘I am busy with masters of midwifery and I am allowed to rotate even on the adult and neonatal ICU which is in relevance to my studies.’ (IP#3, 25 years old, midwife)

## Discussion

In this qualitative study, the perceptions of midwives on shortage and retention of staff were summarised in three main themes as emerged from the data analysis. The three themes were shortage of midwives, reasons for leaving the profession and the reasons for staying in the profession. The midwives expressed the frustrations of working under the conditions of limited staff in a shift as having a negative impact on their workload. These research findings relate to those of Thopola and Lekhuleni (2015:508) that increased workloads result from several factors such as inadequate number of midwives who are on duty and feeder clinics that are not adhering to referral criteria.

Midwives seem to be dissatisfied with the manner in which the referral system is managed. In the study conducted in Limpopo Province of South Africa, it was revealed that shortage of midwives, limited material resources, medico-legal hazards and substandard midwifery care were the factors impeding the provision of optimal midwifery practice in maternity units of public hospitals (Thopola & Lekhuleni 2015:511).

Furthermore, the impossible demands of the midwives’ workload could place them in an ethical dilemma of how to prioritise care (Filby, McConville & Portela 2016:10). In order to remedy the situation, midwives find themselves being obliged to work overtime which consequently affects their provision of quality care.

According to Filby et al. (2016:10), in their research study report concerning African countries, inadequate staffing and working excessive overtime were found to compromise safety for women receiving maternal care as well as midwives.

Filby et al. (2016:4) further assert that moral distress and burnout result in midwives becoming disempowered to provide quality care. Midwives are experiencing low morale in relation to their working conditions. Hildingson and Fenwick (2015:180) assert that paying more attention to establishing healthy work environment where midwives feel recognised and valued is likely to improve midwives’ perceptions of themselves and the quality of their working life. Warriner, Hunter and Dymond (2016:194) point out that stress within the midwifery profession has a negative impact on the health and well-being of individual midwives, as well as the care of women.

Midwives are a pillar of reproductive health programmes, and it is crucial to understand their role in the health system and support them (WHO 2013:804). Like all other healthcare staff, midwives are known to experience higher levels of stress (Pezaro et al. 2015:2). While they may be suffering from their own personal stressors and having to sometimes deal with uncooperative patients and disrespectful doctors, midwives report that they struggle to function professionally.

In all health systems, it is essential for personnel to receive some level of continuous training to ensure personnel development and quality improvement. Some midwives in the study feel left out in matters of personal development as they revealed that they are not awarded opportunities to attend peer symposia, such as annual midwifery symposia held in different provinces in the country. Hauck et al. (2017:47) recommended the importance of professional development support and access to senior midwives within the clinical environment in order to ensure quality and sustainability of midwifery workforce. The utilisation of temporary employed midwives is a positive measure to relieve them of their heavy workloads. The midwives in the study perceive employment of temporary midwives as a burden citing the fact that it still remains their duty to orientate and supervise the temporary employed midwives.

The use of temporary employed midwives has its own disadvantages, as cited in a study conducted in South Africa on the indirect cost of agency nurses including: poor attitudes of agency staff, perceived lack of commitment, disloyalty, unreliability, reluctance to take on ‘extra duties’, time taken on supervision and a perception that they do not have the same ‘culture of caring’ when compared to the permanent staff (Rispel & Moorman 2015:5).

Dixon et al. (2017:6) explored the reasons midwives are leaving the profession and find a range of issues related to following: the working conditions within the national health service; lack of resources; lack of management support; not having control over work; and not having time to develop or sustain the relationships with women and colleagues (Dixon et al. 2017:6). The midwives in this study reported a few reasons similar to the ones cited by Dixon et al. (2017). Although the midwives do not have any intentions to leave the profession, they are likely to leave the institution hoping for better conditions elsewhere because of shortage which leads to stress and burnout. The midwives however are sensitive to the fact that shortage of staff is a universal crisis.

Changing employers may provide a sense of relief from the known unchanging environment. Versaevel (2011:30) suggests that the underlying reasons for midwives leaving include not feeling valued, long working hours, poor organisational culture, lack of support, low morale and increasing struggles with work–life balance

Lack of managerial support as highlighted by participants was one challenge which according to them contributes to the exodus of midwives. In the study conducted in Malawi, the common reason for intention to leave was poor management (Chimwaza et al. 2014:3). According to Chipeta et al. (2016:6) staff retention improves when staff feel supported by the nurse manager and is often linked to manager’s approachability, openness and balance when dealing with problems which arise during work.

The participants commonly agree that they are excluded in many decision-making processes which affect their daily work and would prefer to be involved and own the decisions. Ditlopo et al. (2014:8) emphasise that nurses’ participation in the development of policies and strategies enhances their job satisfaction and retention in the health sector. Through role managing and leading maternity units the midwives can use this knowledge to influence policy and service direction (Bannon, Alderdice & McNeill 2017:659). According to Pezaro et al. (2015:2), midwives could be at an increased risk of work-related psychological stress, because of the fact that they are independent practitioners and working in an area of high litigations. According to Jervis and Choucri (2016:21), midwives are being investigated by their professional bodies for issues resulting from failings in the system, lack of midwifery support or leadership, low staff levels, bullying, policy and protocol, not evidence based, obstetric dominance, lack of funding and lack of services.

Financial challenges are broadly mentioned as contributory factors to job dissatisfaction by many researchers. According to Ditlopo et al. 2013:138, financial incentives are commonly used as a strategy to improve health worker motivation and retention. According to Ditlopo et al. (2013:139), the South African government introduced Occupation Specific Dispensation (OSD) as a financial strategy to attract, motivate and retain health professionals in the public sectors in the clinical specialities; however, numerous problems with the implementation left nurses dissatisfied.

Midwives have a strong belief in the independent function of midwifery and feel that their autonomous status is currently compromised. A number of midwives consulted during the ICM Triennial Congress workshop felt that the status they are given is not as high as that experienced by other members of health profession (WHO 2016b:26). According to the International Confederation of Midwives, the scope of practice for midwives must support and enable autonomous midwifery practice and should therefore include prescribing rights, access to laboratory/screening services and admitting and discharge rights (Oyetunde & Nkwonta 2014:43). The South African Nursing Council has, meanwhile, been criticised over not recognising midwifery as a profession in its own right (Duma et al. 2012:6). This is perhaps the reason why midwifery as an independent function is still undervalued. The challenge of under-defined job descriptions posed more frustrations as midwives are perceived as all-rounders, having to perform all kinds of duties including working with files and serving meals. Too much paperwork is taking the midwives’ time from providing the necessary care to the women, as there is too much repetition of documentation. The obstetric book is a document meant to cover all information pertaining to the woman, from antenatal care to puerperium. More documentation is added according to the participants in the study, such as the ICU observation charts regardless of the woman’s condition in the ward.

The reasons to stay in the profession are both personal and professional. Some midwives are fearful of change and others have no other place to work, while others have a passion for midwifery and cannot imagine deviation from what they know. Midwives are passionate about the care they provide to women and their families and the positive impact they have on the outcome of the pregnancy. They expressed substantial pride in their work, especially when faced with emergency complicated maternal cases such as attending to an eclamptic mother and are able to actually save a life. Training opportunities are cited as the contributory factor for inclination to stay in the profession. Among the factors that promote retention is training opportunities according to Ghapanchi and Aurum (2011) as cited in Kossivi, Xu and Kalgora (2016:262). The midwives working in high dependency obstetrics, as the participants in the study, require knowledge and skills beyond those required to provide care to ‘well’ women (Eadie & Sheridan 2017:6).

According to Asegid, Belachew and Yimam (2014:3), career opportunities and training afford individuals the prospect of further developing themselves and growing within the ranks of their career. A conducive work environment is essential to retain the staff. The participants cited availability of resources as compared to other public hospitals being part of that which keeps them from leaving.

## Strengths and limitations

This study revealed a need for a collaborative effort by management of maternity wards to attract and retain the midwives in the profession through participative management in maternity healthcare services.

The interviews were conducted during working hours because of the availability of participants which might have affected the data collection based on the pressure to prioritise their primary function while participating in the interviews. Most of the participants were not willing to avail themselves for participation while they were off duty stating reasons of fatigue.

## Implications and recommendations

The shortage of midwives is influenced by many factors surrounding the profession itself. This chronic shortage is a compelling factor to midwives performing overtime which has a direct effect on the quality maternity care. Midwives in this research study indicated that the challenges of poor supervisory support have the biggest impact on the midwives’ decision to continue in the profession.

Managers should understand the pressures midwives work under, therefore should not add to the pressure but bring encouragement through positive feedback and constructive criticism.

Communication platforms need to be created in order for managers to listen to the concerns and views of staff. Where policy-making is involved, a representative from the maternity unit should be present to assist in determining the feasibility of implementation considering the status quo.

More flexible retention strategies are needed to retain midwives as opposed to the currently implemented policy in Gauteng Province. To retain midwives, the relevant stakeholders need to create opportunities where midwives could share their feelings and be reassured of the support from management.

The research findings reveal that many students do not choose to practise as midwives because of fear of litigation. The training institutions should conduct a situational analysis not only looking at the availability of material resources and patients, but the human resources, in order to safeguard the welfare and training of students. Further study is therefore recommended in investigating the impact of excessive use of overtime hours on shortage of staff and the quality of midwifery care.

## Conclusion

Midwives are at the forefront of maternity services and need to be perceived by managers of hospitals as such. It is evident that lack of supervisory support affects midwives’ decision to remain or leave the profession. Positive feedback and constructive criticism is important in the development of midwives in their profession. Provision of material and human resources including a good working environment is essential in ensuring the provision of quality services to women and to avoid litigation. Despite the fact that midwifery is included in the four-year course leading to the registration as nurse (general, community and psychiatry) and midwife, it still remains a scarce skill as many after registration do not volunteer to practise as midwives. To attract and retain midwives continues to be a battle which needs collaborative efforts.
